# Recent progress and challenges in the treatment of spinal cord injury

**DOI:** 10.1093/procel/pwad003

**Published:** 2023-02-10

**Authors:** Ting Tian, Sensen Zhang, Maojun Yang

**Affiliations:** Ministry of Education Key Laboratory of Protein Science, Beijing Advanced Innovation Center for Structural Biology, Beijing Frontier Research Center for Biological Structure, School of Life Sciences, Tsinghua University, Beijing 100084, China; Ministry of Education Key Laboratory of Protein Science, Beijing Advanced Innovation Center for Structural Biology, Beijing Frontier Research Center for Biological Structure, School of Life Sciences, Tsinghua University, Beijing 100084, China; Ministry of Education Key Laboratory of Protein Science, Beijing Advanced Innovation Center for Structural Biology, Beijing Frontier Research Center for Biological Structure, School of Life Sciences, Tsinghua University, Beijing 100084, China; Cryo-EM Facility Center, Southern University of Science and Technology, Shenzhen 518055, China

**Keywords:** spinal cord injury, axon regeneration, functional recovery, therapeutic strategies, clinical translation

## Abstract

Spinal cord injury (SCI) disrupts the structural and functional connectivity between the higher center and the spinal cord, resulting in severe motor, sensory, and autonomic dysfunction with a variety of complications. The pathophysiology of SCI is complicated and multifaceted, and thus individual treatments acting on a specific aspect or process are inadequate to elicit neuronal regeneration and functional recovery after SCI. Combinatory strategies targeting multiple aspects of SCI pathology have achieved greater beneficial effects than individual therapy alone. Although many problems and challenges remain, the encouraging outcomes that have been achieved in preclinical models offer a promising foothold for the development of novel clinical strategies to treat SCI. In this review, we characterize the mechanisms underlying axon regeneration of adult neurons and summarize recent advances in facilitating functional recovery following SCI at both the acute and chronic stages. In addition, we analyze the current status, remaining problems, and realistic challenges towards clinical translation. Finally, we consider the future of SCI treatment and provide insights into how to narrow the translational gap that currently exists between preclinical studies and clinical practice. Going forward, clinical trials should emphasize multidisciplinary conversation and cooperation to identify optimal combinatorial approaches to maximize therapeutic benefit in humans with SCI.

## Introduction

Spinal cord injury (SCI) leads to long-term dysfunction and lifelong disability. There are hundreds of thousands of new patients suffering an SCI each year worldwide, and ninety percent of these SCIs are caused by traumatic events, including traffic accidents, falling, sports injuries, violence, etc. (Ashammakhi et al., 2019). SCI interrupts the neural connections between the upper center and the spinal cord, leading to devastating and permanent neurological impairment, including sensory and motor disability, abnormal reflexes, and autonomic disorders (Ehrmann et al., 2020; Ong et al., 2020). SCI not only seriously affects the quality of life and life span of patients, but it also leads to psychological and emotional disorders and social phobia, with enormous impact and heavy economic burden on the family as well as the social health care system (Alizadeh et al., 2019). Therefore, effective treatments are urgently needed to cure individuals with SCI.

The pathophysiology of SCI involves primary injury and secondary injury. The primary injury is caused by acute mechanical trauma and results in vascular disruption, blood–spinal cord barrier rupture, cell death (neurons, glia cells, and endothelial cells), and interruption of neural fiber tracts in the spinal cord. The secondary injury references the consecutive pathological events triggered by the primary injury, such as hemorrhage, excitotoxicity, neuroinflammation, demyelination, astrogliosis and extracellular matrix (ECM) remodeling, which aggravate tissue damage, and compromise neuroplasticity (Rowland et al., 2008). The current clinical treatment for SCI mainly includes early surgical decompression and stabilization, augmentation of spinal cord perfusion, intravenous administration of high-dose corticosteroids, anti-inflammatory therapy in the acute phase, and neurological rehabilitation training during the chronic phase (Karsy and Hawryluk, 2019). Unfortunately, the clinical benefit achieved using current approaches is limited and there are no effective strategies currently existing to repair SCI, thus patients suffer long-term dysfunction and lifelong disability. Axon regeneration and functional recovery after SCI remains one of the most challenging medical problems in the field of neuroscience. In recent years, owing to the development of novel molecular biology, materials science, genetic engineering, and computer science approaches, researchers have developed a variety of novel therapeutic concepts to treat SCI, including tissue engineering, gene editing, and neuromodulation technology. These strategies have shown promising results in preclinical studies, and some have entered phase 1 or 2 clinical trials. However, relatively few inventions have been completed due to lack of clinical efficacy.

In this review, we provide an overview of recent advances and challenges in SCI research and treatment. We summarize the recent progress regarding interventions after SCI in preclinical rodent and non-human primate models as well as in the clinical settings. We then discuss the current status and challenges with respect to clinical translation, prospect the future of SCI repair, and we highlight combinatory approaches and multidisciplinary cooperation are imperative to optimize clinical outcomes after SCI. This review provides an update of current strategies following SCI and provide researchers with new insights to develop more effective and targeted interventions to facilitate clinically meaningful recovery after SCI.

## Intrinsic and extrinsic determinants controlling axon regeneration

**Figure 1.**
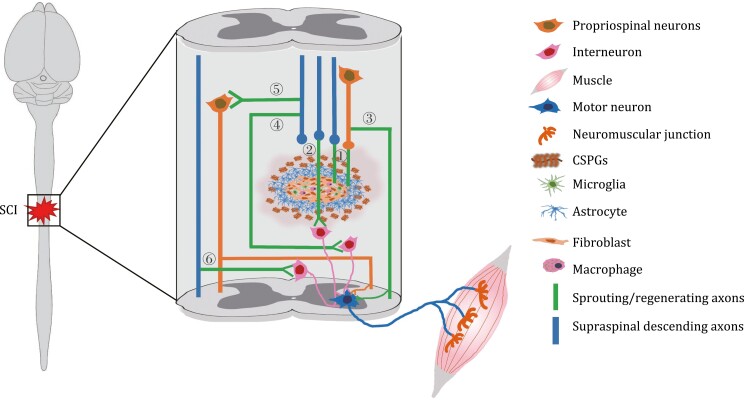
**Neuronal regeneration following spinal cord injury involves multiple forms of axon growth.** (1) The transected axons of supraspinal and propriospinal neurons regrow to pass through the scar borders and towards the lesion core, while failing to move beyond the injury site. (2) Long-distance regeneration of transected supraspinal axons across the lesion site to form functional connections with motor neurons via interneurons. (3) Sprouting of injured propriospinal neurons to bypass the lesion area to reach appropriate targets. (4) The sprouting axons of injured supraspinal neurons bypass the injury site to form new synaptic connectivity with interneurons. (5) The sprouting axons of injured supraspinal neurons make connections with the contralesional spared propriospinal neurons to relay supraspinal commands to motor neural network below the injury site. (6) Sprouting of intact supraspinal axons to cross the midline to regain control over the motor circuits below the lesion. These growth processes support certain degrees of functional restoration.

**Figure 2.**
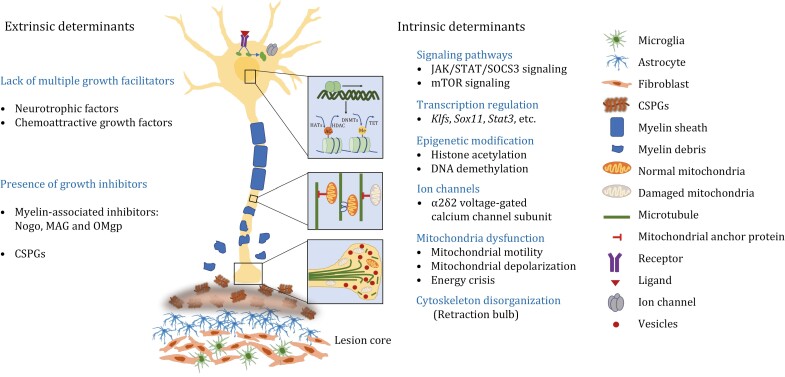
**Cellular and molecular mechanisms determining axon regeneration.** The extrinsic and intrinsic determinants control the intrinsic regenerative ability of injured neurons following SCI.

The originally narrow concept of regeneration referred to the regrowth of transected axons across the lesion core to form functional synapses with their original pre-injury targets after SCI. The broad definition of regeneration now encompasses multiple forms of axon growth, including long-distance axon regrowth, compensatory sprouting of injured and spared supraspinal axons as well as propriospinal neurons, synapse remodeling, and circuit reorganization (Sofroniew, 2018) (Fig. 1). Over the past decades, intensive research efforts have demonstrated that regeneration failure after severe SCI is attributed to poor intrinsic regrowth capacity of adult neurons as well as the non-permissive local environment. Understanding the intrinsic and extrinsic mechanisms determining axon growth and regeneration failure is therefore crucial for designing potential therapeutic strategies (Fig. 2).

### Intrinsic mechanisms underlying axon regeneration

#### Cytoskeletal dynamics

Axon outgrowth is driven by the growth cone, a motile structure located at the tip of the growing axon. Growth cone motility and axon growth are determined by the neuronal cytoskeleton, which includes microtubule and actin filament (F-actin) (Lowery and Vactor, 2009; Blanquie and Bradke, 2018). Microtubules are the structural scaffolds of an axon, and their continuous assembly and remodeling are pivotal for regulating growth cone motility and subsequent axon regrowth (Hur et al., 2012). Actin dynamics in the growth cone play a key role in growth cone pathfinding and axon regeneration (Leite et al., 2021). Enhancing actin polymerization at the leading edge induces growth cone protrusion and extension, whereas promoting F-actin disassembly leads to growth cone collapse and retraction (Lowery and Vactor, 2009).

Cytoskeletal dynamics and rearrangements are mainly modulated by two intracellular signaling pathways: glycogen synthase kinase 3β (GSK3β) and Rho GTPases. GSK3β is downstream of PI3K signaling and is inactivated through serine-9 phosphorylation within its amino-terminal region upon activation of the PI3K pathway. GSK3β has been shown to be a crucial negative regulator of microtubule dynamics by phosphorylating multiple microtubule binding proteins (MBPs), such as collapsin response mediator protein 2 (CRMP-2), adenomatous polyposis coli (APC), and microtubule-associated protein-1B (MAP1B) (Zhou and Snider, 2005). Inhibition of GSK3β activity abolishes phosphorylation of MBPs and promotes microtubule assembly and axon growth (Zhou et al., 2004; Yoshimura et al., 2005). The Rho family of GTPases, which include ras homolog gene family member A (RhoA), Ras-related C3 botulinum toxin substrate 1 (RAC1), and cell division control protein 42 homolog (CDC42), are a class of molecules that modulate cytoskeleton rearrangements to regulate the growth cone. RhoA GTPases integrate upstream pathways and coordinate downstream cytoskeletal effectors (e.g., Rho-associated kinase (ROCK), formins, ENA/VASP, Arp2/3) to modulate actin dynamics, such as actomyosin contraction, F-actin disassembly and polymerization, as well as F-actin treadmilling and retrograde flow (Lowery and Vactor, 2009; Hall and Lalli, 2010). Suppression of RhoA GTPase activity or downstream effectors promotes actin dynamics and subsequent axonal growth (Gwak et al., 2017; Stern et al., 2021).

Enhancing cytoskeletal dynamics in the growth cone supports axon growth, whereas aberrant cytoskeletal dynamics following injury represents a major obstacle to axon regeneration. Therefore, manipulations targeting cytoskeletal dynamics may be potential strategies to induce axon regeneration of adult neurons following injury.

#### Intracellular signaling pathways regulating axon regeneration

##### JAK/STAT/SOCS3

Janus kinase (JAK)/signal transducer and activator of transcription (STAT) signaling is involved in regulating axon regeneration and cell survival. JAK is activated by growth factors and cytokines (e.g., ciliary neurotrophic factor [CNTF] and interleukin 6 (IL-6)) and then phosphorylates STATs, which transduce the signals to the nucleus and promote transcription of regeneration-associated genes (RAGs). Suppressor of cytokine signaling 3 (SOCS3) is a negative regulator of the JAK/STAT pathway. Ablation of *Socs3* in adult retinal ganglion cells magnified JAK/STAT signaling, thereby inducing axon regeneration after optic nerve injury (Smith et al., 2009). Activation of the JAK/STAT pathway via *Cntf* overexpression or *Socs3* deletion is sufficient to stimulate sprouting of corticospinal tract (CST) axons in the mouse spinal cord (Jin et al., 2015).

##### mTOR signaling

The mammalian target of rapamycin (mTOR) pathway plays a crucial role in regulating fundamental cell processes, including cell growth and metabolism, gene transcription, protein synthesis, and cytoskeletal remodeling (Lipton and Sahin, 2014; Saxton and Sabatini, 2017). mTOR function is mediated through two mTOR complexes, known as mTORC1 and mTORC2. mTORC1 is activated by Rheb, the activity of which is suppressed by the tuberous sclerosis complex (TSC). Both PI3K/AKT and RAS-RAF-MEK-ERK can inhibit TSC2 and promote mTORC1 activation, while AMPK and GSK inhibit mTORC1 by increasing TSC activity (Saxton and Sabatini, 2017). Phosphatase and tensin homolog (PTEN) is a negative regulator of PI3K and thus suppresses mTOR activation. Deletion of *Pten* promotes mTOR activation and positively modulates regeneration of facial nerve and CST axons (Du et al., 2015; Meyer Zu Reckendorf et al., 2022). Activation of AKT/mTOR signaling via targeting growth factor receptors or by expressing constitutively active *Rheb* also enhances mTOR activation and axon regeneration (Wu et al., 2015; Zhao et al., 2021b).

##### DLK signaling pathway

Dual leucine zipper-bearing kinase (DLK), a highly conserved mitogen-activated protein kinase kinase kinase (MAP3K), plays a vital role in injury response, apoptosis, axon transport, and regeneration following injury both in the peripheral nervous system (PNS) and in the central nervous system (CNS) (Asghari Adib et al., 2018). Upon axon injury, DLK activates c-Jun N-terminal kinase (JNK) signaling and then initiates transcription of proapoptotic genes and RAGs (Watkins et al., 2013). DLK signaling is essential to initiate axonal regrowth in damaged neurons, and overexpression of *dlk-1* is sufficient to enhance regrowth capacity of injured axons in *C. elegans* (Ko et al., 2020). Germinal cell kinase four (GCK-IV) kinases inhibition promotes axon regeneration of retinal ganglion cells (RGCs) after optic nerve injury via interactions with DLK/leucine zipper kinas (LZK) signaling (Patel et al., 2020).

#### Transcriptional and epigenetic regulation

The limited axon regeneration following injury in the adult CNS is partially owing to poor activation of RAGs. RAG expression is regulated by pro-regenerative transcription factors (TFs), which initiate pro-regenerative transcriptional programs and enable the reacquisition of regenerative potential. Previous studies have identified multiple pro-regenerative TFs, such as Krüppel-like factors (*Klfs*), *Sox11*, *Stat3*, cAMP-response element binding protein (*Creb*), and hypoxia inducible factor-1a (*Hif-1a*) (Lang et al., 2013; Cho et al., 2015; Bradley et al., 2019; Chang et al., 2021; Kramer et al., 2021). Manipulations of these TFs have been reported to improve the regenerative capacity of adult neurons in the CNS following injury.

Epigenetic modifications, which include histone acetylation, DNA demethylation and hydroxymethylation, as well as modification of non-coding RNAs, facilitate access to gene regulatory regions by TFs, thus enabling active transcription of genomic regions and RAG expression. Epigenetic modifications play a key role in embryonic and adult neurogenesis (Yao et al., 2016). In recent years, epigenetic modifications have emerged as a potential target to enhance neuronal plasticity and regeneration after CNS injury (Puttagunta et al., 2014; Weng et al., 2017; Hutson et al., 2019).

### Extrinsic inhibitory components restricting axon regeneration

It is difficult to achieve frank regeneration of injured axons beyond the lesion site in the adult mammalian CNS. Apart from limited intrinsic regenerative competence, multiple extrinsic barriers contribute to the axon regeneration failure. Among these, myelin-associated inhibitors (MAIs) and chondroitin sulfate proteoglycans (CSPGs) represent the major inhibitors to axon regeneration following SCI. Nogo A, myelin-associated glycoprotein (MAG), and oligodendrocyte myelin glycoprotein (OMgp) are three prototypical myelin-associated inhibitory molecules that exert inhibition on axon regeneration after injury in the adult CNS. These MAIs initiate intracellular Rho and ROCK signaling pathways through multiple receptors (e.g., Nogo receptors [NgRs] and PirB) and eventually lead to growth cone collapse and prevent neurite extension (Geoffroy and Zheng, 2014). CSPGs are a large family of ECM molecules secreted by multiple cell types (e.g., astrocytes, oligodendrocyte progenitor cells, stromal cells, and inflammatory cells) and are highly deposited surrounding the scar tissue following SCI. CSPGs are another key group of axon regrowth inhibitors, which activate RhoA/ROCK signaling through receptors including LAR, PTPσ, and NgRs to downregulate neuronal plasticity and impede axon regeneration (Silver and Silver, 2014; Bradbury and Burnside, 2019). Neutralizing these inhibitory factors via pharmacological or genetic manipulations might create a more favorable microenvironment for axon regeneration and has been shown to foster functional recovery after SCI (Lang et al., 2015; Rosenzweig et al., 2019; Schneider et al., 2019; Wang et al., 2020).

## Therapeutic strategies for spinal cord repair

**Figure 3.**
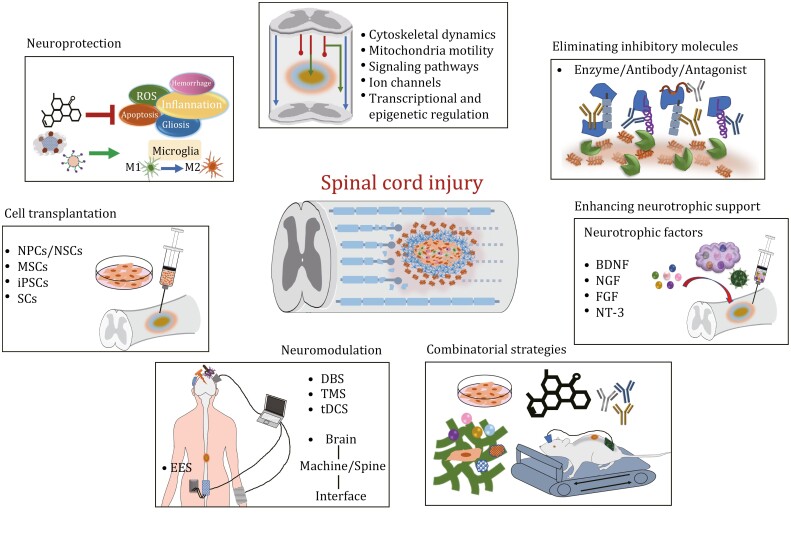
**Therapeutic strategies for spinal cord repair.** The illustration summarizes the promising interventions to improve functional recovery following SCI. Neuroprotective strategies counteract the progression of the secondary injury. Targeting intrinsic regenerative mechanism promotes neuroplasticity and axon regeneration. Eliminating inhibitory molecules and enhancing neurotrophic support provide a growth-permissive environment for axon regeneration. Cell transplantation facilitates the formation of neural relay circuits across the lesion site. Neuromodulation technology enables control over the activity of paralyzed legs and reorganization of spared circuits. Finally, combinatorial strategies contribute to maximize meaningful functional recovery by targeting multifaceted SCI pathology.

Spinal cord injury interrupts the connectivity of the spinal cord and leads to devastating neurological deficits. In addition, concomitant complications, including respiratory and urinary infections, gastrointestinal disorders, muscle atrophy, and chronic pain exacerbate clinical outcomes. Numerous novel approaches have been emerged to ameliorate SCI outcomes that can be divided into two parts: (i) relieving secondary damage via neuroprotection and (ii) fostering neuroplasticity and axon regeneration by triggering intrinsic regenerative mechanisms, ameliorating the extrinsic environment, and applying cell transplantation and neuromodulation technologies (Fig. 3). Each of these strategies will be discussed in the following sections.

### Neuroprotection

The primary mechanical injury to the spinal cord triggers a cascade of complex biological processes, termed the secondary injury, which cause further damage to the spared tissue and exacerbate neurological impairment and regeneration failure. Therefore, interventions that confer neuroprotection during the acute phase are crucial to suppress the spread of the secondary injury and to protect the spinal cord tissue from further damage. Several strategies have been deployed to attenuate the secondary damage. Pharmacological agents like methylprednisolone, minocycline, and cyclosporine A have been used to in patients with SCI to suppress secondary damage (Griffin and Bradke, 2020). However, the application of these drugs remains controversial because none of them has demonstrated beneficial neurologic outcomes in SCI patients, and some even lead to adverse events (Casha et al., 2012; Liu et al., 2019b).

In recent years, a large number of novel neuroprotective approaches have emerged designed to counteract the progression of secondary injury. These therapies have demonstrated neuroprotective effects in preclinical studies and provide new perspectives for the treatment of clinical SCI. Oxidative stress following SCI leads to excess release of reactive oxygen species (ROS), which exacerbates secondary injury and results in axon degeneration and permanent neurological dysfunction. Antioxidant enzymes encapsulated in biodegradable nanoparticles have been shown to significantly reduce ROS activity and neuronal cell apoptosis, attenuate mitochondrial dysfunction, and improve locomotor recovery following severe contusive SCI in rats (Andrabi et al., 2020). In a mouse contusive SCI model, the highly bioactive iridium complex, IrFPHtz, effectively eliminated excessive ROS and suppressed the inflammatory response via interaction with superoxide dismutase 1 (SOD1), thereby facilitating neuronal regeneration and recovery of behavioral function (Ji et al., 2022). ROS can also be scavenged by cerium oxide nanoparticles (CONPs), which reduced pro-inflammatory and apoptotic molecules and improved locomotor function in a rat model of spinal cord contusion (Kim et al., 2017).

Overactivation of microglia after SCI exacerbates the inflammatory response and results in loss of neurons, gliosis, and synaptic damage. Inhibition of microglia proliferation by oral administration of the CSF1R inhibitor, GW2580, reduced neuroinflammation and improved locomotor function in mice and non-human primates following lateral spinal cord hemisection (Poulen et al., 2021). Microglia and macrophages act as key players in modulating neuroinflammation with two different phenotype, pro-inflammatory (M1) phenotype and anti-inflammatory (M2) phenotype. Promoting the anti-inflammatory M2 phenotype of microglia/macrophages after SCI has demonstrated beneficial outcomes in preclinical studies. For example, hyaluronan-based hydrogel loaded with fat extract induced microglia/microphage M2 polarization, which alleviated inflammation, reduced tissue impairment, and promoted motor functional recovery of hindlimbs after compressive SCI in mice (Xu et al., 2022). Similarly, the DAMP-scavenging, IL-10-releasing hydrogel scaffold promoted M2 polarization of microglia/macrophages and axon regeneration and suppressed inflammation following complete thoracic spinal cord transection in mice (Shen et al., 2022). M2 macrophage-derived exosomes loaded with berberine also exhibited anti-inflammatory and anti-apoptotic properties by inducing M2 polarization (Gao et al., 2021). Blood–spinal cord barrier (BSCB) disruption and progressive hemorrhage are the most destructive events following acute SCI. Flufenamic acid (FFA) was recently studied for its potential to prevent secondary hemorrhage and BSCB disruption. FFA effectively decreased microglia activation, cavity formation, reactive gliosis, and myelin loss, and ultimately protected motor neurons and promoted functional restoration in mice after contusive thoracic SCI (Yao et al., 2018). With further development, these neuroprotective interventions that have achieved beneficial outcomes in preclinical investigations can be combined with other pro-regenerative strategies to improve clinical efficacy after acute SCI.

### Targeting neuronal intrinsic regenerative capacity

While self-repair spontaneously occurs after injury in the adult mammalian PNS, mature neurons in the CNS fail to regain regenerative competence after SCI. Axon regeneration failure is mostly attributed to the poor intrinsic growth competence of the adult CNS neurons. Axon growth capacity sharply declines with age and is switched off upon maturation. However, over the last few decades, accumulating evidence suggests that axon regeneration and remodeling of neural circuits are possible via pharmacological, molecular, or genetic manipulations in the adult mammalian CNS (Tedeschi and Bradke, 2017). Substantial investigations have been devoted to reactivating intrinsic growth programs and fostering axon regeneration by modulating the neuronal intrinsic determinants, providing hope for repairing the damaged spinal cord. The intrinsic mechanisms controlling axon regeneration primarily include cytoskeletal dynamics, signaling pathways, ion channels, transcriptional regulation, and epigenetic modification.

#### Cytoskeletal dynamics

Microtubule destabilization and actin disassembly following SCI lead to growth cone collapse and retraction bulb formation. The reformation of a growth cone-like structure from severed axon stumps is the first step in axon regeneration. Most manipulations aiming to augment axon regenerative ability eventually converge on cytoskeletal dynamics and remodeling within the growth cone.

The microtubule-stabilizing drugs Taxol and epothilone B have been shown to induce axon regeneration and functional improvement by enhancing microtubule polymerization and decreasing fibrotic scarring after moderate spinal cord contusion in rodents (Hellal et al., 2011). However, epothilone B was also found to suppress functional recovery after SCI by aggravating inflammation burden (Mao et al., 2017). Activated RhoA following SCI inhibited the functions of CRMP2 and Cofilin via ROCK, leading to growth cone collapse and regeneration failure. Consistently, *Rhoa* knockdown was shown to enhance axon regeneration and reduce apoptosis, cavity formation, and astrogliosis in a rat compression SCI model (Gwak et al., 2017). A recent study revealed that RhoA has opposing roles in different cell types during CNS regeneration. Neuron-specific *Rhoa* inactivation enhanced axon regeneration after dorsal column SCI in mice, whereas astrocyte-specific *Rhoa* deletion hampered functional recovery following SCI, suggesting that cell-specific *Rhoa* manipulation might be a promising therapeutic intervention (Stern et al., 2021). In contrast to microtubule dynamics, the role of actin dynamics in axon regeneration remains poorly understood and deserves further investigation. Tedeschi et al. reported that ADF/cofilin was a key regulator of actin dynamics and played a central role in growth cone motility. Enhanced severing activity of ADF/cofilin promoted actin turnover and was sufficient to drive central axon regeneration of dorsal root ganglia (DRG) neurons after crush injury in the spinal cord of adult mice (Tedeschi et al., 2019).

Cytoskeleton rearrangement and growth cone reformation of injured axons requires high levels of energy in the form of adenosine triphosphate (ATP), which is mainly produced in the mitochondria. SCI-induced mitochondrial dysfunction and energy deficits exacerbate axon regeneration failure. Syntaphilin (Snph) is a static anchor protein that holds axonal mitochondria stationary on microtubules via its microtubule-binding domain. Enhancing mitochondrial transport by *Snph* deletion or increasing energy metabolism via creatine administration contributed to the recovery of the local energy supply, thereby promoting robust axon regeneration, functional synapse reconnection, and motor function restoration after severe SCI in mice (Han et al., 2020). The mitochondria-targeted P21-activated kinase 5 (PAK5) distributes on axonal mitochondrial surfaces and rapidly declines during neuron maturation. Reprogramming the AKT-PAK5 axis enhanced mitochondrial transport by turning off Snph-mediated anchoring, thus reversing the axonal energy crisis and facilitating neuron survival and axonal regenerative sprouting after dorsal spinal cord hemisection in adult mice (Huang et al., 2021).

#### Intrinsic signaling pathways

Intracellular signaling pathways, such as mTOR, JAK/STAT/SOCS3 and DLK regulate neuronal survival and axon growth. Accumulating evidence suggests that manipulating these pathways could enhance intrinsic growth competence and neuroplasticity after SCI.

Decreased mTOR activity upon maturation is a major cause of limited regenerative capacity in adult neurons. In a mouse model of crush SCI, upregulation of mTOR activity by deletion of PTEN, a negative regulator of mTOR, significantly increased the regenerative potential of injured CST axons as well as collateral sprouting of intact CST axons (Du et al., 2015). Deletion of *Socs3* triggered collateral sprouting of intact CST axons to the denervated spinal cord after unilateral pyramidotomy. Moreover, co-deletion of *Pten* and *Socs3* further enhanced CST sprouting with significant restoration of skilled locomotion function, suggesting the reformation of functional circuits mediated by sprouting axons (Jin et al., 2015). Rheb is the upstream of mTOR and directly activates the mTOR pathway. Increasing mTOR activity by expressing constitutively active *Rheb* promoted both sprouting and regeneration of damaged axons following thoracic and cervical SCI in rats (Wu et al., 2015; Urban et al., 2018). It is previously reported that the activity of mTOR was negatively regulated by AMPK. More recently, Giovanni team found that conditional ablation of AMPKα1 elicited the intrinsic regenerative capacity of DRG neurons following spinal dorsal column axotomy in mice. The injured ascending DRG sensory fibers regenerated into and across the lesion site, suggesting AMPK as a crucial regulator of axon regeneration after SCI (Kong et al., 2020).

DLK is a key regulator of neuronal regeneration and becomes activated following the injury response. Activated DLK stimulates downstream signaling pathways, including JNK and p38MAPK, which are implicated in growth cone formation and axon shaft consolidation (Asghari Adib et al., 2018). The role of DLK in regulating axon regeneration after rodent SCI remains relatively unknown. Recently, Saikia et al. (2022) demonstrated that DLK and LZK possessed multicellular function in promoting regeneration and compensatory sprouting of CST axons after spinal dorsal hemisection in mice. Liver kinase B1 (LKB1), a convergent downstream effector of the cAMP/PKA and PI3K signaling pathways, plays a critical role in cell growth and differentiation, and it was shown that *Lkb1* overexpression could promote long-distance regeneration of CST axons and significant locomotion recovery in adult mice with dorsal hemisection (Ohtake et al., 2019).

#### Ion channels

The α2δ2 subunit of voltage-gated calcium channels acts as a developmental switch that positively regulates synapse formation while limiting axon growth and regeneration. Pharmacological blockade of α2δ2 with gabapentinoid promoted regeneration of CST and ascending sensory axons in mice after crush SCI (Tedeschi et al., 2016; Sun et al., 2020). Early administration of gabapentinoids also improved motor outcomes after human SCI (Warner et al., 2017). These findings strongly suggest that gabapentinoids may be a potential pharmacological intervention to enhance neurological recovery after acute SCI. KCC2 is a neuronal-specific chloride potassium cotransporter that maintains low intracellular chloride (Cl^−^) concentration by extruding Cl^−^ from the cytosol. KCC2 has important roles in synaptic inhibition as well as in neuronal development and plasticity (Kaila et al., 2014). KCC2 activation via agonist or overexpression reactivated the dormant relay spinal circuitry and ultimately restored consistent stepping ability in paralyzed mice with staggered bilateral hemisection (Chen et al., 2018a). Downregulation of KCC2 has been associated with SCI-induced spasticity, and enhancing KCC2 activity in the lumbar enlargement decreased hyperreflexia and spastic symptoms in chronic SCI animals (Bilchak et al., 2021). Collectively, these findings support manipulating KCC2 as a promising approach to promote functional recovery and to improve the quality of life for individuals with SCI.

#### Transcriptional and epigenetic regulation

Transcription factors (TFs) directly modulate transcription of RAGs and initiate the switch from the resting state to the robust regrowth state in damaged neurons. TFs have been shown to be an important determinant controlling axon growth potential in the mature CNS. Over the past decades, researchers have identified several candidate TFs that potentiate the regenerative capacity of injured axons after SCI.

Overexpression of *Klf6* or *Klf7* promoted sprouting and regeneration of CST axons in adult mice (Blackmore et al., 2012; Kramer et al., 2021). In addition, *Nr5a2* synergistically enhanced the effect of *Klf6* to augment axon outgrowth. By upregulating the gene modules linked to macromolecule synthesis and DNA repair, combined expression of *Klf6* and *Nr5a2* enhanced collateral sprouting of both spared and injured CST fibers and induced robust extension into the denervated territory in mice after pyramidotomy injury (Venkatesh et al., 2021). Although forced expression of *Klf6* promoted robust cross-midline sprouting of spared CST axons after pyramidotomy in mice, *Klf6*-induced sprouting was insufficient to ameliorate functional deficits of the forelimb (Kramer et al., 2021). CREB signaling is another potential target for axon regeneration and sprouting. Selective inhibition of *Creb*-mediated transcription in spinal relay neurons changed circuit remodeling of CST axons in mice following dorsal hemisection of the spinal cord; specifically, the collateral length of CST axons and the density of synaptic contacts with relay neurons significantly decreased (Bradley et al., 2019). Human placental mesenchymal stem cell (hpMSCs)-derived exosomes improved locomotor and bladder function as well as promoted significant endogenous neurogenesis after rat spinal cord transection. Follow-up mechanistic studies suggested that the endogenous neurogenesis was probably triggered by activation of MEK/ERK/CREB signaling (Zhou et al., 2021). Forced overexpression of *Sox11* in cortical neurons facilitated compensatory sprouting in intact CST axons and promoted axon regeneration in injured CST axons. Unfortunately, *Sox11* overexpression also impaired forelimb dexterity (Wang et al., 2015). However, optogenetic activation of *Sox11*-stimulated CST terminals promoted the formation of functional synapses without deficits in mouse forelimb dexterity following pyramidotomy (Jayaprakash et al., 2016). Lin28a is an RNA-binding protein and acts as a gatekeeper molecule to regulate conversion between somatic cells and pluripotent stem cells. Overexpression of *Lin28a* dramatically triggered long-distance regeneration of corticospinal axons and promoted locomotor functional recovery in adult mice with dorsal over-hemisection (Nathan et al., 2020).

In addition to TFs, gene expression is also regulated by epigenetic mechanisms, such as histone acetylation and DNA demethylation. Histone modifications have previously been shown to be involved in axon regeneration. p300/CBP-associated factor (PCAF) promoted histone 3 Lys 9 acetylation (H3K9ac) following peripheral injury, and *Pcaf* overexpression augmented regeneration of ascending sensory fibers after dorsal column axotomy of the mouse spinal cord (Puttagunta et al., 2014). Recent studies have suggested that Creb-binding protein (Cbp)-mediated histone acetylation could enhance neuronal plasticity and regenerative potential after SCI in rodent models. Also, increasing the activity of Cbp acetyltransferase induced regeneration and sprouting of proprioceptive afferents and descending motor axons, leading to improvement in sensory and motor functions after spinal cord contusion in rodents (Hutson et al., 2019). These results identify Cbp activation as a potential target for clinical SCI treatment. The role of DNA demethylation has also been investigated in axon regeneration. Injury-induced DNA demethylation mediated by thymine DNA glycosylase (TDG) and tet methylcytosine dioxygenase 3 (TET3) elevated the expression of RAGs and supported axon regeneration of adult DRG neurons as well as behavioral improvement (Weng et al., 2017). This study suggests that active DNA demethylation can remove intrinsic epigenetic ‘brakes’ on RAG expression to elicit robust axon regeneration in the adult CNS.

### Ameliorating the local microenvironment

In addition to limited intrinsic growth ability, the hostile microenvironment is another obstacle to axon regeneration after SCI. Indeed, extrinsic growth-inhibitory factors and the deficiency of neurotrophic factors further hamper neuroplasticity and axon regeneration. Therefore, removing inhibitory molecules and augmenting neurotrophic support contribute to render a more permissive environment and promote functional recovery following SCI.

#### Eliminating inhibitory barriers

The presence of inhibitory factors (Nogo A, MAG, OMgp, CSPGs, etc.) surrounding the lesion site appears to be the major environmental obstacle to axon growth that prevents the injured axons from real regeneration. Removal of extrinsic inhibitory components by enzymatic degradation, receptor blocking, or antibody neutralization has demonstrated positive effects on axon regeneration and neurological restoration.

##### Targeting CSPGs

Highly concentrated CSPGs within the lesion scar present a barrier to axon regeneration after SCI (Bradbury and Burnside, 2019). Mitigating the inhibitory role of CSPGs may therefore be an effective therapeutic intervention to enhance axon regeneration following SCI. Degradation of CSPGs through enzyme digestion is a known potential solution for spinal cord repair. A single injection of chondroitinase ABC (ChABC) was sufficient to restore robust and persistent respiratory motor function up to 1.5 years after cervical SCI in rats. Combining ChABC and intermittent hypoxia conditioning further enhanced the ventilatory response (Warren et al., 2018). Moreover, tight temporal control of *Chabc* gene expression was shown to promote recovery of skilled reaching and ladder walking performance as well as sensory axon conduction following cervical contusion injury in adult rats (Burnside et al., 2018). Four weeks after spinal cord hemisection in rhesus monkeys, multiple parenchymal injections of ChABC below the lesion significantly enhanced collateral sprouting of spared CST axons and restored dexterous hand control with no detectable detrimental effects, supporting the potential for clinical translation of ChABC treatment (Rosenzweig et al., 2019). Another enzyme that can degrade CSPGs is arylsulfatase B (ARSB), which selectively removes inhibitory C4S GAGs via hydrophobic interaction. Sustained release of ARSB using a dual-functional hydrogel system enhanced recovery of locomotor function, which was accompanied by regeneration of serotonergic and propriospinal axons after rat contusive SCI (Park et al., 2022).

The CSPG-specific signaling receptors, PTPσ and LAR, are promising targets for blocking the effects of CSPGs. PTPσ has a critical role in CSPG-mediated inhibition by converting growth cones into a dystrophic state. Daily subcutaneous injection of intracellular sigma peptide (ISP) to specifically modulate PTPσ released the inhibitory effect of CSPGs on axon growth and markedly fostered functional recovery of sensory, locomotor, and urinary systems after contusive SCI in rats (Lang et al., 2015). Inhibition of LAR has been shown to substantially relieve CSPG-mediated inhibition. LAR inhibitory peptide significantly promoted functional diaphragm recovery and robust regeneration of injured rostral ventral respiratory group (rVRG) axons as well as sprouting of spared rVRG and serotonergic axons after cervical hemisection in rats (Cheng et al., 2021). Inhibition of PTPσ and LAR receptors supported oligodendrogenesis, oligodendrocyte differentiation, maturation, and myelination, and attenuated oligodendrocyte apoptosis in a rat model of compressive SCI (Dyck et al., 2019). Additionally, blocking of LAR and PTPσ receptors activated a supportive and beneficial immune response after compressive SCI, and their inhibition elevated pro-regenerative M2 microglia/macrophages with a remarkable increase in anti-inflammatory mediators (Dyck et al., 2018).

##### Targeting MAIs

Attenuating MAI-mediated inhibition can be achieved by antibody neutralization, receptor antagonists, and genetic inhibition. Anti-Nogo A treatment ameliorated lower urinary tract dysfunction and restored bladder function in rats with severe SCI (Schneider et al., 2019). The first-in-man investigation of a human anti-Nogo A antibody, ATI355, demonstrated that ATI335 was safe and well tolerated in patients with acute traumatic paraplegia and tetraplegia. Further trials are warranted to evaluate the efficacy of ATI355 to improve functional recovery following SCI (Kucher et al., 2018).

The human NgR1-Fc effectively blocked NgR1 and promoted locomotor recovery in multiple preclinical rodent models of SCI (Wang et al., 2014). The safety and efficacy of NgR1-Fc has also been demonstrated in non-human primates. In cynomolgus monkeys subjected to C5/C6 right hemisection, NgR1-Fc facilitated behavioral recovery and regeneration of CST axons without evident toxicity (Wang et al., 2020). Lateral olfactory tract usher substance (LOTUS) is an endogenous NgR1 antagonist. *Lotus* overexpression has been proved to enhance axon regeneration of the raphespinal tract and reticulospinal tract fibers, suppress axonal dieback of CST fibers, and promote motor recovery in a mouse contusive SCI model (Ito et al., 2018). Additionally, overexpression of *Lotus* in human induced pluripotent stem cell-derived neural stem/progenitor cells enhanced survival and axonal extension of the transplanted cells, and significantly promoted motor functional recovery after contusive SCI in mice (Ito et al., 2021). Administration of a LINGO-1 antagonist contributed to axonal sprouting and enhanced functional recovery of rat forelimb after dorsal or lateral hemisection of the spinal cord (Ji et al., 2006). *Lingo-1* deletion mitigated cell apoptosis, inflammatory response, and glial scar inflammation after complete spinal cord transection in mice (Huang et al., 2019). *Lingo-1* inhibition by miR-615 promoted axonal regeneration and remyelination and improved functional recovery of hindlimbs in rats subjected to complete SCI (Wu et al., 2020). Furthermore, *Lingo-1* shRNA enhanced neuronal differentiation of neural stem and progenitor cells (NSPCs) into neurons and oligodendrocytes, and transplantation of *Lingo-1* shRNA-treated NSPCs facilitated locomotor recovery in mice with contusive SCI (Zhao et al., 2021a). Overall, these preclinical and clinical studies suggest that interventions targeting MAIs can augment neuroplasticity and axon regeneration following SCI.

#### Enhancing neurotrophic support

Neurotrophic factors (NFs) are a family of proteins that direct neuronal survival, synaptic function, and axonal growth within the adult nervous system (Keefe et al., 2017). NFs are substantially reduced after SCI, and the lack of NFs within the local environment is another cause for poor axon regeneration and functional recovery. Therefore, increasing the level of NFs can provide a more permissive environment to trigger pro-regenerative responses and ameliorate dysfunction following SCI. NFs can be delivered by multiple injections, continuous infusion, or released via viral vector and biological materials.

Brain-derived neurotrophic factor (BDNF) is a secreted protein that fosters neuron survival and synaptic plasticity. Delivery of BDNF mRNA with cationic polymers resulted in improved motor function recovery in a mouse model of contusive SCI (Crowley et al., 2019). Intraspinal injection of an AAV2-*Bdnf* vector into the lesion significantly enhanced sprouting of 5-HT^+^ axons and increased their synaptic connections with phrenic motor neurons (PhMNs), and contributed to recovery of diaphragm function following cervical SCI (Charsar et al., 2019). Retrograde transport of neurotrophin-3 (NT-3) via AAV delivery system to lumbar MNs reinforced propriospino-MN circuit reorganization and restored lumbar motor circuitry, leading to improved behavioral and electrophysiological recovery after T9 spinal cord contusion in mice (Han et al., 2019). Sustained release of NT-3 from fibroin scaffolds reduced inflammatory response, promoted regeneration and remyelination of nerve fibers, and improved locomotion performance as well as electrophysiological recording in canine SCI (Li et al., 2018a). In addition, effective delivery of neuronal growth factor (NGF) with a nanocapsule-based delivery system inhibited the inflammatory response and facilitated neuronal regeneration and significant functional recovery in a mouse model of compressive SCI. This delivery technology opens up a new avenue for the treatment of CNS injuries (Xu et al., 2019). Insulin-like growth factor 1 (IGF-1) plays an important role in both developmental and pathological conditions. In a mouse model of moderate contusive SCI, overexpression of *Igf-1* reduced endothelial apoptosis and hemorrhage area, and enhanced behavioral recovery via activation of PI3K/AKT pathway (Li et al., 2020). *Igf-1* overexpression in bone mesenchymal stem cells also improved cell survival and promoted remyelination and functional improvements after spinal cord contusion in mice (Allahdadi et al., 2019). Subcutaneous administration of fibroblast growth factor 2 (FGF2) decreased inflammation, gliosis, and CSPGs, and ultimately improved functional recovery of the paralyzed hindlimb in mice after spinal cord hemisection (Goldshmit et al., 2014). In addition, orthotopic injection of FGF13 in the rat spinal cord after crush injury demonstrated beneficial effect on axon regeneration by regulating microtubule dynamics and mitochondrial function (Li et al., 2018b). Vascular endothelial growth factor (VEGF) is a key regulator of angiogenesis while platelet-derived growth factor (PDGF) promotes mature vessel formation. In rats with hemisection injury of the spinal cord, combination of VEGF and PDGF reduced cavity size and gliosis, and ameliorated inflammatory response, creating a more permissive microenvironment for axon regeneration (Lutton et al., 2012). VEGF was also found to facilitate the activation of neural stem cells after complete SCI in rats via VEGF-VEGFR2-EGFR signals, providing new insights for maximizing endogenous neurogenesis following SCI (Liu et al., 2019a).

### Cell-based therapies

The substantial loss of neurons and oligodendrocytes following SCI leads to disruption of neural network and signal transduction. Cell transplantation exhibits multitherapeutic capacities, such as replacement of damaged tissue, formation of relay neural circuits, immunomodulation, neuroprotection, and myelin regeneration, thus emerging as a more promising intervention to promote functional improvement following SCI (Assinck et al., 2017). A variety of cell types, including NSPCs, induced pluripotent stem cells (iPSCs), MSCs, Schwann cells (SCs), and olfactory ensheathing cells (OECs) have been used to promote spinal cord repair.

Neural stem cells/neural progenitor cells (NSCs/NPCs) are self-renewing cells and can differentiate into specific neurons or glial cells to replace damaged spinal cord tissue. Implantation of NPCs/NSCs has shown great therapeutic potential in axon regeneration and functional restoration after SCI in rodents and primates (Lu et al., 2012; Rosenzweig et al., 2018). The observed functional benefits may derive from functional synapse formation between the host and graft that generate neuronal relays across the lesion site. After NPC transplantation to the injured spinal cord, the regenerating adult axons maintained the capacity to recognize appropriate neuronal targets within the NPC grafts and re-establish complex neural circuitry in rats after dorsal column lesion of the spinal cord (Dulin et al., 2018). A subsequent study demonstrated that the grafted NPCs spontaneously segregated into distinct domains resembling the normal spinal cord. The regenerating CST axons preferentially located the motor domains of NPC grafts and formed functional neuronal connections without additional exogenous guidance (Kumamaru et al., 2019). In mice with dorsal column spinal cord lesions, the NSCs-derived neurons integrated into the host circuitry and could trigger local host neuronal network below the lesion; Meanwhile, the NSCs-derived neurons could be activated by host motor and sensory inputs, demonstrating the restoration of trans-lesion synaptic connectivity (Ceto et al., 2020). The phase I clinical trial of human NSC (NSI-566) transplantation for the treatment of chronic complete SCI has been completed. The procedure was well tolerated by all participants and no serious adverse events were detected within the post-procedure follow-up period. Furthermore, the neurological function of two subjects was partially improved, supporting NSI-566 as a safe approach with potential to yield positive clinical outcomes in patients with SCI (Curtis et al., 2018). In another study, 6-year follow-up data demonstrated long-term safety and preliminary efficacy of human central nervous system stem cells (HuCNS-SC) when transplanted in 12 subjects with traumatic motor complete SCI. Although no patient achieved motor functional recovery of the lower limbs, 5 of the 12 patients acquired subtle and stable sensory improvements with no tumor formation and no deterioration of spinal lesion area (Curt et al., 2020).

MSCs are ideal candidate for cell-based therapies. MSCs raise no ethical concerns and can be acquired from diverse tissue sources like bone marrow, placenta, umbilical cord, and adipose tissue. Therefore, MSC transplantation is one of the most promising candidates for SCI repair. The therapeutic effects MSC transplantation after SCI are primarily due to their paracrine activity and trophic support: MSC-secreted factors modulate the immune response and induce neuroprotection, angiogenesis, and fiber regeneration in the injured spinal cord (Papa et al., 2018; Cao et al., 2022). In the subacute phase of rat incomplete SCI, intrathecal transplantation of human umbilical cord-derived mesenchymal stem cells (hUC-MSCs) improved locomotor performance and promoted electrophysiological recovery. The underlying molecular mechanisms was associated with restoration of the excitation/inhibition balance in injured spinal neurons through upregulating GABAAR subunits and KCC2 (Cao et al., 2022). Transplantation of 3D human placenta-derived MSCs (3D-HPMSCs) to the damaged spinal cord exhibited remarkable effects on neuroprotection and alleviated axonal dieback in mice subjected to spinal cord hemisection. 3D-HPMSCs also demonstrated better functional recovery on electrophysiological conduction, locomotion, limb coordination, and trunk stability compared to 2D-HPMSCs (Deng et al., 2021). These encouraging results from preclinical rodent SCI studies provide the theoretical basis for clinical trials of MSC transplantation.

SCs are another potential candidate for transplantation in patients with SCI. SCs have been shown to promote spinal cord repair through multiple mechanisms; they myelinate axons, reduce tissue injury, enhance neuroprotection, and maintain axonal plasticity. SCs are easily acquired from autologous nerve, thus alleviating the risk of immune rejections and avoiding the need for immunosuppression. SCs have demonstrated potential therapeutic effects in rodent SCI models, and their safety and potential efficacy have also been confirmed in clinical trials. A phase I clinical trial in humans with subacute SCI suggested that autologous human Schwann cell (ahSC) transplantation injected into the epicenter of the spinal lesion was safe and feasible. No serious adverse events or neurological complications were detected within in one year after transplantation (Anderson et al., 2017). Another phase I clinical trial of ahSC transplantation was completed in participants with chronic SCI. When combined with multimodal rehabilitation, transplantation of ahSCs isolated from sural nerve improved motor and sensory function without adverse events, again indicating the safety and feasibility profiles of ahSC transplantation (Gant et al., 2022). Co-transplantation of autologous MSCs and SCs during the subacute period of SCI in patients significantly improved sensory and motor functions. Patients achieved remarkable recovery in trunk movement, posture equilibrium, and voluntary voiding with no systemic complications (Oraee-Yazdani et al., 2021). Another phase 1 clinical trial of ahSC transplantation in six participants with complete subacute SCI demonstrated neurophysiological changes between 6 and 12 months after transplantation. However, no clinical improvements were observed, and longer observation may be required to determine whether ahSC transplantation is clinically effective to repair SCI (Santamaria et al., 2021).

Like ahSCs, iPSCs derived from somatic cells avoid ethical concerns and immune rejection, and they can be reprogrammed to NPCs/NSCs, supporting human iPSC-derived NSC/NPC transplantation as a viable approach to treat SCI. Transplantation of human iPSC-derived NSCs facilitated axon growth and functional neural circuit reconstruction as well as motor function restoration after cervical spinal cord hemisection in rats (Lu et al., 2014). Similarly, implantation of NSCs/NPCs with gliogenic competence from human iPSC promoted axon remyelination and motor function recovery after contusive SCI in mice without tumor formation in the lesion area (Kamata et al., 2021). However, after transplantation, undifferentiated iPSCs are thought to pose a safety risk, such as promoting tumorigenesis. One approach to address this issue is the selective deletion of immature proliferating cells by herpes simplex virus 1 thymidine kinase (HSVtk), which was shown to prevent tumorigenesis after hiPSC-NS/PC implantation without sacrificing the regained motor function after contusive SCI in mice (Kojima et al., 2019).

Collectively, the multiple observed therapeutic benefits associated with cell transplantation makes cell-based therapy an attractive approach that could substantially improve outcomes for patients with SCI. However, some remaining issues may limit their clinical practice, including ethical controversy, cell source, intervention time, uncontrolled cell proliferation, and long-term safety. Continued efforts to resolve these issues and concerns could make cell transplantation a feasible clinical option.

### Neuromodulation-based interventions

The descending motor pathways are severely interrupted after SCI. Although the spinal neural circuits below the injury level remain intact, the absence of supraspinal descending commands leads to the circuits functionally dormant and loss of motor function. Neuromodulation is a bioengineering approach that utilizes electrical or magnetic stimulation, pharmacological agents, optogenetics, and chemogenetics to modulate neuronal activity. Neuromodulation has been successfully applied in a variety of neurological diseases. Neuromodulatory technologies applied for SCI aim to reactivate the spinal intrinsic motor circuits below the lesion and ultimately restore the basic motor function and/or voluntary movements. These neuromodulatory inventions primarily include spinal cord stimulation, brain stimulation, and brain–machine interface.

#### Spinal cord stimulation

Spinal cord stimulation is emerging as a potential therapy to alleviate neurological deficits and promote functional recovery following SCI. Epidural electrical stimulation (EES), transcutaneous spinal cord stimulation (tcSCS) and intraspinal microstimulation (ISMS) are the three forms of spinal cord stimulation that designed to facilitate functional restoration after SCI.

Epidural electrical stimulation (EES) applied to the lumbosacral spinal segments has been shown to substantially restore locomotion and motor control after SCI both in preclinical and clinical studies. Paralyzed rats treated with EES were able to perform continuous stepping, walking, and even climbing staircases (Wenger et al., 2014). Co-delivery of EES and monoamine receptor agonists to lumbosacral segments immediately restored cortical control over the paralyzed legs after severe spinal cord contusion in rats. When combined with gravity-assisted rehabilitation, this electrochemical neuromodulation triggered extensive cortico-reticulo-spinal circuit reorganization, and enabled cortical control of leg movements without the need of neuromodulation in the long-term (Asboth et al., 2018). Lumbosacral EES combined with rehabilitation training enabled independent standing and assisted walking, and promoted the recovery of voluntary leg movement in people with complete lower extremity paralysis (Angeli et al., 2014). In a human with complete paraplegia after SCI, with 43 weeks of dynamic task-specific training, EES of the lumbosacral spinal networks allowed independent bilateral stepping on a treadmill, and also enabled independent standing and even stepping over ground with the assistance of a front-wheeled walker and trainer to maintain balance (Gill et al., 2018). Four patients with motor complete paralysis achieved independent standing and trunk stability after treatment with EES and intense gait training. Additionally, two of four patients regained the ability to walk independently over ground (Angeli et al., 2018).

Continuous EES may abolish proprioceptive information in humans and restrict locomotion recovery. In contrast, spatiotemporal EES enhanced the active control of motor neurons while preserving the interaction between antagonistic muscles to ensure coordination and stability of lower limb movement (Formento et al., 2018). Spatiotemporal EES achieved voluntary control over paralyzed limbs during overground walking in individuals with chronic cervical SCI. Participants gained a greater degree of independence in outdoor activities like walking and cycling with spatiotemporal EES and maintained neurological recovery even without stimulation (Wagner et al., 2018). More recently, a paddle-leads-with-optimized-electrode arrangement was designed to enhance EES coverage and selectivity. With this neurotechnology, participants with complete sensorimotor paralysis regained their ability to stand, walk, cycle, swim, and control trunk movements independently in ecological settings, demonstrating superior efficacy in restoring motor functions after severe SCI (Rowald et al., 2022). In addition, cervical EES restored arm and hand control in macaque monkeys following cervical SCI, providing a pathway for the development of EES technologies to facilitate voluntary movement of arm and hand in people with quadriplegia (Greiner et al., 2021). Furthermore, EES inventions may also help to improve cardiovascular, urinary, and other physiological functions (Walter et al., 2018; West et al., 2018; Willyard, 2019).

tcSCS is another well-developed form of spinal cord stimulation and has been demonstrated to restore motor, sensory, and autonomic function when used alone or combined with other interventions (Benavides et al., 2020). tcSCS improved the movement control of the paralyzed extremity after SCI. Participants with chronic complete paraplegia regained self-assisted standing and postural control of upright sitting by receiving tcSCS (Sayenko et al., 2019). In addition, tcSCS promoted cardiovascular recovery and significantly ameliorated autonomic dysreflexia in rats after complete spinal cord transection (Sachdeva et al., 2021). More recently, tcSCS restored voluntary control of hand and arm movements in participants with chronic cervical SCI. The regained function was sustained for months, indicating long-term functional recovery mediated by neuroplasticity. Additionally, tcSCS improved autonomic functions and relieved muscle spasticity (Inanici et al., 2021). Compared to EES, tcSCS is more attractive to clinical application as it is non-invasive, inexpensive and easily available. Unlike tcSCS, ISMS delivers electrical stimulation with surgically implanted electrodes within the spinal cord and is mainly applied in SCI animal models. In rats with cervical contusive SCI, the delivery of activity-dependent ISMS led to markedly behavioral recovery of the impaired forelimb. The achieved performance could be maintained for weeks beyond electrical stimulation (McPherson et al., 2015). A wireless ISMS system successfully evoked graded and sustained activation of multiple synergistic muscles and produced repeatable hindlimb movements in rats with complete SCI (Grahn et al., 2015). Using a silicon-based 3D microelectrode, ISMS alleviated bladder dysfunction and promoted voiding efficacy in one cat with spinal cord transection (Pikov et al., 2020). ISMS could also effectively evoke diaphragm motor units after upper cervical hemisection in rats (Mercier et al., 2017). Although ISMS are not yet suitable for application in humans currently, the advantage of selectively stimulating specific components within the spinal neural networks makes it a potential approach for restoring functional control after SCI.

#### Brain stimulation

Previous studies have indicated that even clinically complete SCI spares some descending nerve fibers, but they are dormant and insufficient to trigger movement. Brain stimulation has been used to engage the residual motor circuits to restore voluntary limb movement after SCI. The brain stimulation strategies mainly include deep brain stimulation (DBS), transcranial direct current stimulation (tDCS), and transcranial magnetic stimulation (TMS).

DBS is a well-established technique that activates neural activity of the target brain region via implanted electrode. Motor cortex electrical stimulation was shown to promote CST axon sprouting, and the combination of motor cortex stimulation and transcutaneous spinal direct stimulation (tsDCS) further augmented CST axon plasticity and restored forelimb motor function after rat spinal cord contusion (Zareen et al., 2018; Yang et al., 2019). This combined therapy also enhanced rehabilitation efficacy and improved skilled locomotion after cervical contusion in rats (Sharif et al., 2021). Unlike DBS, tDCS is a non-invasive technique of brain stimulation to regulate neuronal activity in the motor cortex. tDCS has been shown to mitigate neuropathic pain in patients with SCI (Yang and Chang, 2021). tDCS with rehabilitation training augmented adaptive plasticity and promoted recovery of upper limb function in participants with incomplete tetraplegia (Potter-Baker et al., 2017). tDCS also improved respiratory function of patients with difficult weaning and led to successful decannulation when associated with respiratory exercise (De Carvalho et al., 2021). TMS is another non-invasive technique of brain stimulation that has been applied in SCI. Repetitive TMS therapy promoted neuroplasticity and improved locomotor performance when delivered in the acute phase after rat contusive SCI, suggesting early TMS intervention might lead to better functional recovery (Krishnan et al., 2019). TMS delivered in combination with peripheral nerve stimulation enhanced hand motor output in patients with chronic tetraplegia (Tolmacheva et al., 2017).Repetitive TMS reduced spasticity symptoms and ameliorated motor function of lower and upper extremity muscles in patients with incomplete SCI, and neural transmission within spinal pathways was also slightly improved (Leszczynska et al., 2019; Wincek et al., 2021). These non-invasive approaches (tDCS and TMS) are relatively safe and low costing, making them attractive clinical options for SCI repair.

#### Brain–machine interface

Brain–machine interface (BMI) has emerged as a novel technique that combines engineering, computer science, and neurophysiology to restore sensorimotor functions in patients with severe neurological disability (Lebedev and Nicolelis, 2017). BMI records and decodes brain activity, extracts the information, and then translates the signals to produce functional outputs by controlling external devices. Over the past decade, BMI has been widely investigated in animal models and clinical trials and has been found to effectively promote neurological recovery in patients with tetraplegia or paraplegia.

A participant with quadriplegia from cervical SCI regained cortical control of dexterous hand movements using BMI. The participant’s forearm muscles were reactivated and controlled by the intracortically recorded signals, ultimately enabling complete functional movement task like grasping, manipulating, and releasing objects (Bouton et al., 2016). An individual with chronic tetraplegia achieved reaching and grasping movements with his paralyzed arm and hand through brain-controlled muscle stimulation (Ajiboye et al., 2017). Intracortical BMI restored brain-to-text communication via handwriting in a participant whose hand was paralyzed and entirely non-functional following SCI. This participant also achieved typing speeds that exceeded any other reported outcomes and were comparable to healthy individuals among the same age group. The typing speeds exceed any other BCI previously reported and were comparable to healthy individuals among the same age groups (Willett et al., 2021). Two participants with tetraplegia achieved independent digital communication at home using wireless intracortical BMI (Simeral et al., 2021). The sense of touch is a key component for multiple aspects of motor control, and a participant with C5 quadriplegia who used closed loop demultiplexing BMI achieved full restoration of touch perception in his hand and simultaneous improvement in motor function (Ganzer et al., 2020).

The wireless brain–spine interface was employed to directly link the intended motor states decoded from leg motor cortex activity to spatiotemporal EES over the lumbosacral segment to re-establish voluntary control of leg activity. This approach alleviated gait deficits and enabled weight-bearing locomotion on a treadmill and overground in rhesus monkeys with unilateral corticospinal tract lesion during the first week post-injury (Capogrosso et al., 2016). As observed in non-human primates, the proportional brain–spine interface allowed overground walking and staircase climbing after rat spinal cord contusion. The brain-controlled modulation augmented locomotor recovery during motor rehabilitation (Bonizzato et al., 2018). Brain–computer–spinal interface also improved recovery of upper limb function in rats after cervical SCI (Samejima et al., 2021). Overall, brain–machine/spine interface provides a powerful tool for functional restoration in humans with SCI.

Neuromodulation therapies have achieved unexpected efficacy in restoring locomotion and volitional control in both SCI animal models and individuals with SCI. Therefore, these neuromodulatory technologies might be the most promising approach to achieve meaningful functional recovery following SCI.

### Combinatorial strategies

Individual treatments are insufficient to restore meaningful locomotion due to the complex pathological responses after SCI. Therefore, combinatory approaches targeting diverse aspects of SCI pathology are warranted to facilitate functional recovery in humans with SCI. Biomaterials serve as physical and structural supports to guide axon outgrowth and have been widely applied in the treatment of SCI. Meanwhile, biomaterials act as a key component of combinatorial approaches, as they provide temporary storage substrates for the long-term release of drugs, bioactive molecules, and transplanted cells to modulate inflammation, neural plasticity, axon regeneration, and cell survival (Kiyotake et al., 2022).

Combined delivery of multiple growth factors and chemokines released from hydrogel depots elicited robust propriospinal axon regrowth after complete SCI in both mice and rats. The regenerating propriospinal axons formed synapse-like contacts with neurons below the lesion site and restored electrophysiological conduction across lesions (Anderson et al., 2018). The supramolecular peptide fibril scaffold that integrated two distinct biological signals has shown promising potential to repair SCI. These bioactive scaffolds promoted cell survival, angiogenesis, and axon regrowth and led to remarkable functional recovery of hind limbs four weeks after severe contusive SCI in mice (Álvarez et al., 2021). Dai group combined poly (propylene fumarate) (PPF) scaffolds with collagen biomaterial and neurotrophin-3 with an engineered collagen-binding domain (CBD-NT3) to build a novel biocompatible delivery system. This combinatorial treatment attenuated the inhibitory microenvironment, enhanced axonal regeneration and synapse formation, and promoted electrophysiological and locomotor improvements following spinal cord transection in rats (Chen et al., 2018b). Synergistic release of the anti-inflammatory drug MPSS and growth factors from the hydrogel depot attenuated cystic cavity and scar formation, protected spared axons, enabled neural circuit reorganization, and subsequently improved functional recovery of the hindlimb locomotion in rats with spinal cord contusion (Ye et al., 2021). Furthermore, the combination of electroconductive hydrogels and bone marrow stem cell-derived exosomes synergistically promoted axonal regeneration and recovery of hindlimb motor function via immunoregulation and remyelination after spinal cord hemisection in mice (Fan et al., 2022).

Previous studies have suggested that dissociated cells injected into the lesion site might be washed away by blood flow and poorly survived due to the inhibitory microenvironment. Therefore, cell implantation alone is insufficient to fill the lesion cavity and integrate into a functional network. To enhance cell survival, growth, and integration at the lesion site, NSCs were embedded into fibrin matrices containing a growth factor cocktail. Co-implantation of NSCs and fibrin matrices induced robust CST axon regeneration within and beyond spinal cord lesion sites, and formed new relay circuits, resulting in improved skilled forelimb function in murine models of SCI (Kadoya et al., 2016). Similarly, transplantation of human NPCs and fibrin matrices into the lesioned spinal cord of rhesus monkeys triggered CST and 5-HT axon regeneration, functional synapse formation, and functional restoration of forelimb following C7 hemisection in the spinal cord (Rosenzweig et al., 2018). Implantation of 3D biomimetic printed scaffolds loaded with NPCs enhanced host axon regeneration, including corticospinal, rubrospinal, and reticulospinal axons after complete SCI in rodents. The formation of neural connections between the host and grafted NPCs restored synaptic transmission across the lesion and ultimately supported motor functional recovery (Koffler et al., 2019). Encapsulation of human iPSCs within a porcine omentum-based hydrogel provided a dynamic, permissive microenvironment for functional implant assembly and supplied biochemical cues for axon regeneration. Indeed, iPSC-derived 3D neuronal networks showed a synergistic effect and significantly improved structural integrity and recovery of behavioral functions after both acute and chronic SCI in mice (Wertheim et al., 2022). *Lycium barbarum* oligosaccharide (LBO) promoted microglia towards M2 polarization. Combination of LBO, nasal mucosa-derived mesenchymal stem cells, and fibronectin hydrogel synergistically enhanced the M2 polarization of microglia and promoted axon remyelination and recovery of hind limb movement after complete spinal cord transection in rats (Yu et al., 2021). Co-inhibition of LAR and PTPσ receptors had a beneficial effect on long-term survival and differentiation of engrafted NPCs into the injured spinal cord and facilitated their synaptic integration within the spinal cord network. This combinatorial therapeutic strategy synergistically improved neurologic recovery without altering pain sensitivity in a rat model of compressive SCI (Hosseini et al., 2022).

## Current status and challenges for clinical translation

Over the past decades, researchers have developed a variety of therapeutic strategies to repair or reactivate the damaged neural circuits following SCI. Many promising approaches with encouraging outcomes in rodent models of SCI are progressing towards clinical trials. Unfortunately, some preclinical strategies are problematic and do not demonstrate relevant efficacy when translated into clinical practice, and some even lead to side effects. Most clinical translations discontinued due to lack of efficacy and have largely failed. Therefore, the road towards meaningful clinical translation of preclinically validated therapeutic concepts remains long and arduous.

Conventional pharmacological agents targeting inflammation have exhibited little effect on SCI and are also associated with potential adverse effects. The novel application of granulocyte colony-stimulating factor (G-CSF) was shown to promote functional recovery in rodent models of SCI, and its safety and feasibility were confirmed in preliminary phase I/IIa clinical trial. However, the phase 3 clinical trial failed to show significant benefit of G-CSF in the primary end point (Koda et al., 2021). A few of the regenerative strategies have progressed into clinical trials, including anti-NogoA antibody and cell-based therapies. At present, phase I/IIa clinical trials have been completed and exhibited certain functional improvements without obvious adverse reactions (Curtis et al., 2018; Kucher et al., 2018). Although ChABC has not been entered into clinical trials, preclinical studies in dogs and rhesus monkeys have reported satisfactory results, indicating great potential for clinical application of ChABC. Neuromodulatory interventions like EES and BMI have shown relevant efficacy in clinical trials in recent years and might be the most promising approaches for functional restoration after SCI. EES on the spinal cord restored voluntary control of the trunk and legs, and enabled standing, walking, cycling, and swimming in ecological settings in people with severe SCI (Wagner et al., 2018; Rowald et al., 2022). A participant with tetraplegia regained voluntary movement of upper limbs through BMI (Ajiboye et al., 2017). Neuromodulation technologies open up new avenues for restoring neurological function and supporting daily activities in humans with SCI. However, it is impossible to achieve widespread application of these approaches in the short term. The potential safety concerns, affordability, availability, as well as the biocompatibility and durability of the electrodes remain obstacles to be overcome to improve accessibility of neuromodulation technologies in the future.

Although encouraging results have been achieved in SCI animal models, interventions that are effective preclinically demonstrate little efficacy to produce functional improvements when translated into clinical trials. Therefore, the beneficial outcomes from rodent models are not always reproduced in clinical practice. One possible reason is the limitation of rodent models for predicting efficacy in human SCI. The anatomy and size of the spinal cord vary greatly between rodents and primates. For instance, the anatomical characteristics and function of descending pathways involved in motor control (e.g., CST) demonstrates pronounced interspecies differences between rodents and primates. In rodents, the CST fibers mostly travel in the dorsal columns of the spinal cord, and no direct connections exist between CST fibers and the cervical motoneurons. By contrast, in humans and non-human primates, the CST fibers are mainly located in the lateral columns and have developed direct connections with motoneurons (Lemon, 2008). In addition, the repair mechanisms and response of motor circuits following SCI are markedly different in rodents versus primates (Friedli et al., 2015). Although rodent-based SCI models are most widely used and essential for developing treatments in SCI, non-human primate models are more suitable to design clinically relevant therapies and evaluate the therapeutic benefits, given that the anatomical structure and pathological outcomes after SCI are more similar to those in humans. Therefore, it is essential to evaluate the effects of interventions in various animal models in preclinical studies to better predict the potential efficacy and limitations for human application.

Future clinical trials for the treatment of SCI should be designed after considering several factors that may influence outcomes. Accumulating more data and comprehensive preclinical testing is essential before initiating clinical trials to minimize the risk of patients experiencing adverse effects or undergoing futile treatment. Moreover, the lack of reproducibility and robustness in preclinical experiments is another major issue contributing to the failure of clinical translation (Schaffran and Bradke, 2019). Thus, it is necessary to replicate the preclinical findings by independent, third-party laboratories before the treatments progress into clinical evaluation. The efficacy and long-term safety of therapies also need to be thoroughly assessed in early phases of clinical trials. Larger-scale and randomized clinical trials are essential to comprehensively evaluate the efficacy and adverse effects associated with interventions for SCI repair. In addition to motor disability, sensory deficit and autonomic disorders directly affect the quality of life after SCI. Therefore, it is advisable to thoroughly evaluate the potential impact of the therapies on sensory, autonomic, and cognitive functions in relevant SCI animal models.

Individual therapeutic interventions are inadequate to resolve the multifaceted pathological processes that occur after SCI and to promote spinal cord repair. Multi-targeted, combinatorial approaches are expected to maximize therapeutic effect. However, this becomes more complicated because many aspects must be taken into account when different strategies are combined. For instance, the optimal temporal window of treatment and whether the treatments are safe, tolerable, and feasible in combination must be determined to identify the most effective synergistic strategies. To optimize combinatorial strategies, determine analytical frameworks and achieve clinically relevant recovery in patients with SCI, it is therefore essential to enhance collaboration and communication between basic researchers and clinicians.

## Conclusion and perspectives

Spinal cord injury interrupts the descending motor pathways and ascending sensory fibers within the spinal cord, leading to multi-system dysfunction and permanent disability. To alleviate the devastating effects of SCI, researchers have developed a variety of novel therapeutic approaches in recent years, some of which have shown promising therapeutic efficacy in preclinical studies and are progressing to clinical trials. This review summarizes the recent progress of preclinical and clinical strategies in SCI treatment and discusses the challenges and prospects for spinal cord repair.

Despite encouraging results in preclinical experiments, many interventions have failed to translate to clinical application. This translational failure is partially due to the complex and multifaceted features of SCI pathology. Hence, a better understanding of the pathological processes following SCI and thorough investigation of intrinsic and extrinsic mechanisms controlling axon growth are urgently warranted to develop novel strategies to achieve greater beneficial outcomes. Notably, most individual therapeutic strategies targeting a single pathophysiological mechanism are insufficient to achieve relevant efficacy, resulting in clinical translation failure. Combinatorial strategies that act on multiple pathological processes of SCI have been shown to be more effective than individual treatments alone. Therefore, combinatorial approaches are more promising to wok synergistically to overcome multiple regeneration barriers and foster neural repair in patients with SCI. Furthermore, multidisciplinary collaboration and conversations are essential to ensure the accuracy of therapeutic design and predictive analysis.

Neuronal regeneration or the formation of relay neural circuits after SCI is not necessarily accompanied by associated functional improvement. Rehabilitation training could augment the adaptive plasticity of residual pathways and is expected to trigger reorganization and integration of neural circuitry to promote longer-lasting functional readouts. Together with appropriate rehabilitation, combinatorial strategies might further enhance recovery of neurological function in clinical SCI. Furthermore, it is vital to select appropriate animal models for preclinical studies, and potential strategies should be extensively investigated preclinically in diverse SCI animal models to validate their therapeutic effects. Pronounced species divergence exists between primates and rodents or carnivores, and non-human primate models are particularly relevant for designing interventions and validating their therapeutic potential before translated to clinical application. In the future, further investigation is warranted to identify the neural regenerative mechanisms of functional recovery after SCI, especially in non-human primate models, as they are more similar to those in humans. These studies might be crucial to develop novel therapeutic interventions targeting particular neural circuits to improve function restoration.

## Abbreviations

3D-HPMSCs: 3D human placenta-derived mesenchymal stem cells
ahSC: autologous human Schwann cell
APC: adenomatous polyposis coli
ARSB: arylsulfatase B
ATP: adenosine triphosphate
BDNF: brain-derived neurotrophic factor
BMI: brain–machine interface
BSCB: blood–spinal cord barrier
CBD-NT3: neurotrophin-3 with an engineered collagen-binding domain
LBO: lycium barbarum oligosaccharide
Cbp: creb-binding protein
CDC42: cell division control protein 42 homolog
ChABC: chondroitinase ABC
CNS: central nervous system
CNTF: ciliary neurotrophic factor
CONPs: cerium oxide nanoparticles
CREB: cAMP-response element binding protein
CRMP-2: collapsin response mediator protein 2
CSPGs: chondroitin sulfate proteoglycans
CST: corticospinal tract
DBS: deep brain stimulation
DLK: dual leucine zipper-bearing kinase
DRG: dorsal root ganglia
ECM: extracellular matrix
EES: epidural electrical stimulation
F-actin: actin filaments
FFA: flufenamic acid
FGF2: fibroblast growth factor 2
GCK-IV: germinal cell kinase four
G-CSF: granulocyte colony-stimulating factor
GSK3β: glycogen synthase kinase 3β
H3K9ac: histone 3 Lys 9 acetylation
HIF-1a: hypoxia inducible factor-1a
hpMSCs: human placental mesenchymal stem cells
HSVtk: herpes simplex virus 1 thymidine kinase
hUC-MSCs: human umbilical cord-derived mesenchymal stem cells
HuCNS-SC: human central nervous system stem cells
IGF-1: insulin-like growth factor 1
IL-6: interleukin
iPSCs: induced pluripotent stem cells
ISMS: intraspinal microstimulation
ISP: intracellular sigma peptide
JAK: janus kinase
JNK: c-Jun N-terminal kinase
KLFs: Krüppel-like factors
LKB1: liver kinase B1
LOTUS: lateral olfactory tract usher substance
LZK: leucine zipper kinas
MAG: myelin-associated glycoprotein
MAIs: myelin-associated inhibitors
MAP1B: microtubule-associated protein-1B
MAP3K: MAPK kinase kinase
MAP3K: mitogen-activated protein kinase kinase kinase
MBPs: microtubule binding proteins
MSCs: mesenchymal stem cells
mTOR: mammalian target of rapamycin
NFs: neurotrophic factors
NGF: neuronal growth factor
NgRs: Nogo receptors
NSPCs: neural stem and progenitor cells
NT-3: neurotrophin-3
OECs: olfactory ensheathing cells
OMgp: oligodendrocyte myelin glycoprotein
PAK5: P21-activated kinase 5
PCAF: p300/CBP-associated factor
PDGF: platelet-derived growth factor
PhMNs: phrenic motor neurons
PNS: peripheral nervous system
PPF: poly (propylene fumarate)
PTEN: phosphatase and tensin homolog
RAC1: ras-related C3 botulinum toxin substrate 1
RAGs: regeneration-associated genes
RGCs: retinal ganglion cells
RhoA: ras homolog gene family member A
ROCK: Rho-associated kinase
ROS: reactive oxygen species
rVRG: rostral ventral respiratory group
SCI: spinal cord injury
SCs: Schwann cells
Snph: syntaphilin
SOCS3: suppressor of cytokine signaling 3
SOD1: superoxide dismutase 1
STAT: signal transducer and activator of transcription
tcSCS: transcutaneous spinal cord stimulation
tDCS: transcranial direct current stimulation
TDG: thymine DNA glycosylase
TET3: tet methylcytosine dioxygenase 3
TFs: transcription factors
TMS: transcranial magnetic stimulation
TSC: tuberous sclerosis complex
VEGF: vascular endothelial growth factor
